# Report on outcomes of valve-in-valve transcatheter aortic valve implantation and redo surgical aortic valve replacement in the Netherlands

**DOI:** 10.1007/s12471-021-01608-0

**Published:** 2021-08-09

**Authors:** G. J. van Steenbergen, B. van Straten, K. Y. Lam, D. van Veghel, L. Dekker, P. A. Tonino

**Affiliations:** 1 Catharina Heart Centre, Catharina Hospital, Eindhoven, The Netherlands; 2 Netherlands Heart Registration, Utrecht, The Netherlands; 3 Department of Biomedical Technology, Eindhoven University of Technology, Eindhoven, The Netherlands

**Keywords:** Cardiology, Cardiothoracic surgery, ViV-TAVI, Redo-SAVR, Real-world outcomes

## Abstract

**Objective:**

We sought to investigate real-world outcomes of patients with degenerated biological aortic valve prostheses who had undergone valve-in-valve transcatheter aortic valve implantation (ViV-TAVI) or reoperative surgical aortic valve replacement (redo-SAVR) in the Netherlands.

**Methods:**

Patients who had undergone ViV-TAVI or redo-SAVR for a degenerated biological aortic valve prosthesis in the Netherlands between January 2014 and December 2018 were eligible for this retrospective study. Patients with a prior homograft, active endocarditis or mechanical aortic valve prosthesis were excluded. Patients were matched using the propensity score. The primary endpoint was a composite of 30-day all-cause mortality and in-hospital postoperative stroke. Secondary endpoints were all-cause mortality at different time points, in-hospital postoperative stroke, pacemaker implantation and redo procedures within one year. Baseline characteristics and outcome data were collected from the Netherlands Heart Registration.

**Results:**

From 16 cardiac centres, 653 patients were included in the study (374 ViV-TAVI and 279 redo-SAVR). European System for Cardiac Operative Risk Evaluation I (EuroSCORE I) was higher in ViV-TAVI patients (19.4, interquartile range (IQR) 13.3–27.9 vs 13.8, IQR 8.3–21.9, *p* < 0.01). After propensity score matching, 165 patients were matched with acceptable covariate balance. In the matched cohorts, the primary endpoint was not significantly different for ViV-TAVI and redo-SAVR patients (odds ratio 1.30, 95% confidence interval 0.57–3.02). Procedural, 30-day and 1‑year all-cause mortality rates, incidence of in-hospital postoperative stroke, pacemaker implantation and redo procedures within one year were also similar between cohorts.

**Conclusion:**

Patients with degenerated aortic bioprostheses treated with ViV-TAVI or redo-SAVR have similar mortality and morbidity.

**Supplementary Information:**

The online version of this article (10.1007/s12471-021-01608-0) contains supplementary material, which is available to authorized users.

## What’s new?

- Patients who had undergone valve-in-valve transcatheter aortic valve implantation (ViV-TAVI) had a higher risk profile than patients after reoperative surgical aortic valve replacement (redo-SAVR).
- Despite the higher risk score, differences in adjusted clinical outcomes of mortality and morbidity were non-significant between ViV-TAVI and redo-SAVR.

## Introduction

Stenotic aortic valve disease is one of the most common valve diseases in Western countries [1]. It is present in roughly 5% of 65-year-olds and the prevalence increases even further with advancing age [1]. Globally, surgical aortic valve replacement (SAVR) was the standard treatment for severe stenotic aortic valve disease until the introduction of transcatheter aortic valve implantation (TAVI) [2]. Initially, TAVI was used in inoperable patients and patients at very high surgical risk [3]. In more recent years, evidence is mounting that TAVI may be non-inferior to SAVR with respect to midterm outcomes in intermediate- and low-surgical-risk patients [4, 5]. Patients with lower surgical risk are frequently younger and the long-term durability of surgical bioprosthetic valves has been well established, while similar long-term follow-up data of TAVI patients are not yet available [5–7].

Transcatheter aortic valves are also biological valves and it is hypothesised that their durability, similarly to that of surgical bioprosthetic valves, diminishes with decreasing age of the recipient at implantation due to structural valve degeneration [6]. If patients outlive the durability of the transcatheter prosthetic aortic valve, future reoperations are guaranteed. Redo-SAVR can be performed with an operative mortality of approximately 4.5% and a low rate of stroke, vascular complications and postoperative aortic regurgitation [8]. Valve-in-valve TAVI (ViV-TAVI) is emerging as a minimal invasive alternative to redo-SAVR. However, comparing clinical outcomes of ViV-TAVI and redo-SAVR patients with a degenerated bioprosthetic aortic valve are scarce,[9] and guidelines are therefore lacking. More evidence is essential for optimal decision-making for patients with stenotic biological aortic valve prostheses, as well as for the current debate on the potential expansion of TAVI indications to patients with lower surgical risk.

In this retrospective study, we investigated all-cause mortality and morbidity (stroke, pacemaker implantation and redo procedures) of consecutive patients who had undergone ViV-TAVI or redo-SAVR in the Netherlands, based on data from the Netherlands Heart Registration (NHR; *Nederlandse Hart Registratie*).

## Methods

### Patient selection

Patients were eligible for this study if they had undergone ViV-TAVI or redo-SAVR for a degenerated biological aortic valve prosthesis in the Netherlands between January 2014 and December 2018. Concomitant coronary procedures—percutaneous coronary intervention (PCI) or coronary artery bypass grafting (CABG)—during ViV-TAVI or redo-SAVR were permitted. Patients with a prior homograft, active endocarditis, mechanical aortic valve prosthesis, emergency (could not wait until the following day) and/or salvage procedure (lifesaving) were excluded. The decision for treatment allocation (ViV-TAVI or redo-SAVR) was made at the discretion of the individual institutions based on deliberation by the Heart Team.

We used data from the NHR, which had been collected by 16 Dutch heart centres. The NHR facilitates nationwide, value-based outcome monitoring of cardiology and cardiac surgery patients for most Dutch heart and PCI centres since 2011 [10]. The selection of outcomes in the registry and their definitions are based on modified Delphi procedures with clinical and non-clinical leaders and patient representatives. The robust methodology of the NHR in outcome selection, collection and analysis results in valid datasets that are suitable for research purposes [11].

### Outcomes of interest and definitions

The primary endpoint of the study was a composite of all-cause 30-day mortality and in-hospital postoperative stroke. Secondary individual endpoints were procedural, 30-day and 1‑year all-cause mortality, in-hospital postoperative stroke, 30-day pacemaker implantation, 30-day major vascular complications and 1‑year rate of redo procedures of the aortic valve. Patients that did not complete 1‑year follow-up were excluded from the 1‑year outcome assessment.

Stroke was defined as permanent neurological dysfunction caused by acute infarction of cerebral, spinal or retinal tissue diagnosed by a consultant neurologist [12, 13]. Mortality was defined as all-cause mortality confirmed by the Personal Records Database (*Basisregistratie Personen*), which is monitored by the Dutch government [13]. Procedural mortality encompassed all-cause mortality from the start of the procedure up to the first three postoperative days. Surgical risk was assessed using the logistic European System for Cardiac Operative Risk Evaluation (EuroSCORE) as proposed by Roques et al. (EuroSCORE II was not available for the period of interest) [13, 14]. Other baseline characteristics and outcome data were defined according to NHR definitions, which are aligned with the updated Valve Academic Research Consortium (VARC-2) criteria [13, 15].

### Statistical analysis

Patient characteristics and primary and secondary outcomes are expressed as means with corresponding standard deviation (SD) or median with interquartile range (IQR) for continuous normal and non-normal distributed data, respectively. Categorical data are presented as absolute and relative frequencies. Student’s *t*-test, Mann-Whitney U test or χ^2^ test was used to make a comparison between the treatment allocation where appropriate.

Due to significant differences in baseline characteristics and the nonrandomised design of the study, propensity-score matching and inverse probability of treatment weighting (IPTW) were used to assess outcomes. The propensity score included all baseline characteristics listed in Tab. 1. For each patient who had undergone redo-SAVR, a propensity score-matched patient who had undergone ViV-TAVI was selected (1:1) using nearest neighbour (with a calliper width of 0.2 of the pooled SD of the logit of the propensity score) and no replacement. Covariate balance was evaluated with standardised mean differences, and a standardised mean difference < 0.1 was considered a negligible difference between cohorts.

**Table 1:** Baseline characteristics of unmatched ViV-TAVI and redo-SAVR patients

Variable	ViV-TAVI (*n* = 374)	Redo-SAVR (*n* = 279)	*P*-value	SMD
Male	215 (57.5)	165 (59.1)	0.69	0.034
Age, years	79 (74–83)	70 (65–74)	< 0.01	1.07
BMI, kg/m^2^	25.7 (23.5–28.7)	26.8 (24.1–29.5)	0.12	0.13
NYHA class III–IV	210 (66.0)	95 (43.4)	< 0.01	0.33
Left ventricular ejection fraction, %	55 (40–55)	55 (45–57)	0.012	0.17
Logistic EuroSCORE I	19.4 (13.3–27.9)	13.8 (8.3–21.9)	< 0.01	0.37
Urgent procedure	65 (17.4)	75 (26.9)	< 0.01	0.23
Diabetes mellitus	75 (20.1)	46 (16.7)	0.31	0.083
Chronic pulmonary disease	71 (19.0)	32 (11.5)	< 0.01	0.21
Previous stroke	44 (11.8)	16 (7.4)	0.092	0.11
Procedure before 2016	219 (58.6)	125 (44.8)	< 0.01	0.28

Data are presented as *n* (%) or median (interquartile range)

*Viv-TAVI* valve-in-valve transcatheter aortic valve implantation, *SAVR* surgical aortic valve replacement, *SMD* standardized mean difference,* BMI* body mass index, *NYHA* New York Heart Association, *EuroSCORE* European System for Cardiac Operative Risk Evaluation

Kaplan-Meier survival curve was used to display the trend in all-cause 1‑year mortality of matched patients. Univariate logistic regression was used to identify predictors of the primary endpoint using unmatched patients. Predictors with a *p*-value ≤ 0.1 in univariate analysis were selected for a multivariate logistic regression model with the primary endpoint as the dependent variable (exact date of event was not available for stroke). Treatment allocation was included and EuroSCORE I was excluded as an independent variable in the multivariate logistic regression model irrespective of the *p*-value in univariate analysis.

A *p*-value < 0.05 was considered significant. Analyses were performed using SPSS 25 (SPSS Inc., Chicago, IL, USA) and RStudio version 1.4.1103 (RStudio Inc., Boston, MA, USA).

## Results

In the 16 cardiac centres in the Netherlands participating in the NHR, a total of 989 patients had undergone an intervention for a degenerated aortic valve prosthesis. After applying the aforementioned exclusion criteria, 653 patients were included in the study. ViV-TAVI had been performed in 374 patients and redo-SAVR in 279 patients.

### Baseline characteristics

In the unmatched cohort, the patients who had undergone ViV-TAVI were significantly older (79 years, IQR 74–83 vs 70 years, IQR 65–74, *p* < 0.01), more frequently experienced New York Heart Association class III–IV symptoms (66.0% vs 43.4%, *p* < 0.01), more often had chronic pulmonary disease (19.0% vs 11.5%, *p* < 0.01) and had a lower left ventricular ejection fraction (55%, IQR 40–55 vs 55%, IQR 45–57, *p* = 0.012) than redo-SAVR patients. The logistic EuroSCORE I was significantly higher in ViV-TAVI patients (19.4, IQR 13.3–27.9) than in redo-SAVR patients (13.8, IQR 8.3–21.9, *p* < 0.01).

For the redo-SAVR cohort, the majority of the patients had a degenerated stented valve (*n* = 234), whereas the remaining 45 patients had an unstented valve at the time of the reoperation. In the ViV-TAVI cohort, pre- and postballoon dilation during the procedure was performed in 96 and 49 patients, respectively. Standardised mean differences indicated covariate imbalance between the study cohorts (Tab. 1). Propensity score matching generated 165 pairs of patients and an acceptable covariate balance was achieved after IPTW (Tab. 2).

**Table 2:** Baseline characteristics of matched ViV-TAVI and redo-SAVR patients

Variable			Propensity score matching		IPTW	
	ViV-TAVI (*n* = 165)	Redo-SAVR (*n* = 165)	*P*-value	SMD	*P*-value	SMD
Male	96 (58.2)	100 (60.6)	0.74	0.049	0.73	0.034
Age, years	74 (70–79)	73 (69–77)	0.11	0.23	0.65	0.044
BMI, kg/m^2^	26.6 (24.1–30.1)	26.7 (24.2–28.8)	0.48	0.060	0.98	0.003
NYHA class III–IV	95 (57.6)	90 (54.5)	0.66	0.061	0.94	0.007
Left ventricular ejection fraction, %	55 (44.5–55)	55 (43–55)	0.74	0.042	0.92	0.010
Logistic EuroSCORE I	17.3 (11.2–23.1)	15.5 (9.3–22.5)	0.14	0.042	0.94	0.007
Urgent procedure	35 (21.2)	41 (24.8)	0.51	0.086	0.95	0.006
Diabetes mellitus	34 (20.6)	29 (17.6)	0.58	0.077	0.95	0.006
Chronic pulmonary disease	28 (17.0)	27 (12.7)	0.35	0.12	0.70	0.038
Previous stroke	15 (9.1)	17 (10.3)	0.85	0.041	0.89	0.013
Procedure before 2016	80 (49.5)	80 (49.5)	1.00	< 0.01	0.79	0.025

Data are presented as *n* (%) or median (interquartile range)

*Viv-TAVI* valve-in-valve transcatheter aortic valve implantation, *SAVR* surgical aortic valve replacement, *IPTW* inverse probability of treatment weighting, *SMD* standardized mean difference,* BMI* body mass index, *NYHA* New York Heart Association, *EuroSCORE* European System for Cardiac Operative Risk Evaluation

### Outcomes

In the unmatched cohort, the primary composite endpoint of 30-day all-cause mortality and in-hospital postoperative stroke was similarly distributed between the ViV-TAVI and redo-SAVR group (6.1% vs 6.7%, *p* = 0.74). In the matched cohort, the primary endpoint also did not show a significant difference between the treatment modalities (odds ratio (OR) 1.30, 95% confidence interval (CI) 0.57–3.02; after IPTW: OR 1.23, 95% CI 0.57–2.65; Tab. 3).

**Table 3:** Clinical outcomes of ViV-TAVI versus redo-SAVR patients

	Unmatched cohort			Propensity score–matched cohort				IPWT-matched cohort	
	ViV-TAVI (*n* = 374)	Redo-SAVR (*n* *=* 279)	*P*-value	ViV-TAVI (*n* = 165)	Redo-SAVR (*n* = 165)	OR (95% CI)	*P*-value	OR (95% CI)	*P*-value
*Primary endpoint*									
Mortality or stroke at 30 days	21 (6.1)	19 (6.9)	0.74	7 (4.2)	14 (8.9)	1.30 (0.57–3.02)	0.53	1.23 (0.57–2.65)	0.59
*Secondary endpoints*									
*Mortality*									
Procedural	9 (2.4)	3 (1.1)	0.25	2 (1.8)	3 (1.2)	0.49 (0.07–2.57)	0.42	0.53 (0.12–2.30)	0.40
30-day	17 (4.6)	14 (5.0)	0.85	5 (6.1)	10 (3.0)	1.63 (0.53–5.50)	0.40	1.23 (0.51–2.95)	0.65
1-year	47 (12.5)	25 (12.0)	0.43	17 (10.3)	17 (10.3)	0.78 (0.38–1.56)	0.48	1.17 (0.64–2.15)	0.61
Pacemaker implantation at 30 days	21 (5.9)	8 (6.5)^a^	0.83	9 (5.5)	7 (4.2)	1.65 (0.53–5.62)	0.39	1.11 (0.42–2.91)	0.83
In-hospital postoperative stroke	7 (2.0)	5 (1.8)	1.00	2 (1.2)	4 (2.4)	1.34 (0.29–6.90)	0.70	0.75 (0.16–3.44)	0.71
Redo procedure at 1 year	5 (1.9)	2 (1.7)^a^	1.00	3 (1.2)	2 (1.8)	1.54 (0.62–4.03)	0.36	1.15 (0.53–2.53)	0.73
Major vascular complication at 30 days	24 (7.5)	NA	–	11 (6.7)	NA	–	–	–	–

Data are presented as *n* (%). Missing values were excluded listwise

*Viv-TAVI* valve-in-valve transcatheter aortic valve implantation, *SAVR* surgical aortic valve replacement, *IPTW* inverse probability of treatment weighting, *OR* odds ratio, *CI* confidence interval, *NA* not applicable

^a^ Missing values ~50% because variable is not obligatory for submission to registry

**Fig. 1:**
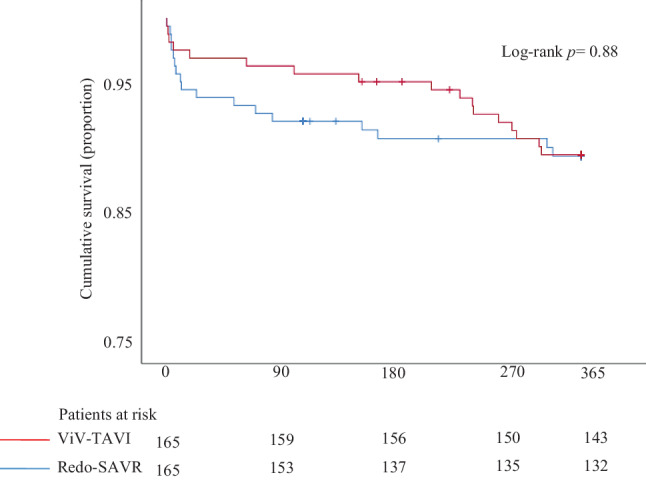
Survival curve of all-cause mortality one-year after valve-in-valve transcatheter aortic valve implantation (*ViV-TAVR*) or redo surgical aortic valve replacement (*SAVR*)

Procedural (2.4% vs 1.1%, *p* = 0.25), 30-day (4.6% vs 5.0%, *p* = 0.85) and 1‑year (11.9% vs 10.0%, *p* = 0.52) all-cause mortality rates (Fig. 1), in-hospital postoperative stroke (2.0% vs 1.8%, *p* = 1.00), 30-day pacemaker implantation rate (5.9% vs 6.5%, *p* = 0.83) and 1‑year redo procedures (1.9% vs 1.7%, *p* = 1.00) did not differ significantly between ViV-TAVI and redo-SAVR patients in the unmatched cohort. In the IPTW-matched cohort, procedural, 30-day and 1‑year all-cause mortality rates (OR 0.53, 95% CI 0.12–2.30 vs OR 1.23, 95% CI 0.51–2.95 vs OR 1.17, 95% CI 0.64–2.15), incidence of in-hospital postoperative stroke (OR 0.75, 95% CI 0.16–3.44), pacemaker implantation at 30 days (OR 1.65, 95% CI 0.53–5.62) and redo procedures at 1 year of follow-up (OR 1.54, 95% CI 0.62–4.03) were also similar between the cohorts. Occurrence of major vascular complications in the ViV-TAVI group was 6.7% in the matched cohort but was not reported in redo-SAVR patients (Tab. 3).

Multivariate logistic regression revealed that an urgent versus elective procedure (OR 2.95, 95% CI 1.52–5.74) and the presence of diabetes mellitus (OR 2.42, 95% CI 1.21–4.86) were significant independent predictors of the primary endpoint (data not shown).

## Discussion

The results of this national registry-based study indicates that in propensity score-matched cohorts, ViV-TAVI for patients with degenerated biological aortic valve prostheses has similar mortality and morbidity as redo-SAVR. This is the largest multicentre study to report consecutive ViV-TAVI and redo-SAVR patients in the Netherlands.

These findings are in corroboration with the general trend described in earlier reports regarding comparative analysis of ViV-TAVI and redo-SAVR. In a pooled analysis of six observational studies (254 patients in total), the authors concluded that ViV-TAVI is possibly be a safe and feasible alternative to redo-SAVR based on absence of statistically significant differences in 30-day mortality (OR 0.91, 95% CI 0.39–2.13) and midterm all-cause mortality (180 days through 3 years; OR 1.42, 95% CI 0.82–2.46).[16] In a larger study population of 717 matched patients, ViV-TAVI conferred similar rates of midterm all-cause mortality (10.9% vs 9.5%, *p* = 0.23) and in-hospital postoperative stroke (1.0% vs 0.4%, *p* = 0.22) compared with redo-SAVR [17]. Hirji et al. compared over 2000 propensity score-matched ViV-TAVI and redo-SAVR patients and also found similar rates of in-hospital postoperative stroke between the cohorts (OR 1.25, 95% CI 0.43–3.64) [18].

In addition, based on the largest meta-analysis to date (8340 patients), Al-Abacha et al. concluded that midterm all-cause mortality (mean 1.7 years, OR 1.15, 95% CI 0.93–1.43) and 30-day pacemaker implantation (OR 0.75, 95% CI 0.42–1.34) were similar between ViV-TAVI and redo-SAVR patients [19]. Interestingly, Al-Abacha et al. described a lower procedural and 30-day mortality rate in favour of ViV-TAVI patients (OR 0.41, 95% CI 0.18–0.96 vs OR 0.58, 95% CI 0.45–0.74), which the authors attributed to the invasiveness of redo-SAVR combined with comorbidities and high surgical risk [19]. Nevertheless, diversity of the included populations and the retrospective design of the included studies may have introduced significant bias.

Despite the seemingly good performance in terms of all-cause mortality and morbidity of ViV-TAVI in comparison with redo-SAVR in our and other studies, several challenges need to be considered prior to potentially expanding primary TAVI indications to a younger patient population. First, lack of evidence on valve durability of TAVI in general remains a concern. Prospective long-term follow-up data of primary TAVI in low-surgical-risk patients does not exceed 5 years [7]. Midterm outcomes in terms of haemodynamic performance from a continued access registry as part of the prospective PARTNER II trial are promising and recently showed that the reduction in mean gradient and the increased effective orifice area after ViV-TAVI is sustained up to 3 years [20, 21]. Haemodynamic performance data with longer follow-up are not yet available, but 38% of the patients who underwent ViV-TAVI in 2014 were still alive after 8 years [22].

For surgical bioprostheses, long-term durability and the influence of age and other patient factors have been well established and redo-SAVR yields good patient outcomes [6]. Furthermore, residual gradient and patient-prosthesis mismatch are relevant issues that can likely be attributed to underexpansion of the TAVI valve [18]. Especially patients with small bioprostheses (< 21 mm) are challenging in terms of haemodynamic performance after ViV-TAVR [23]. Interesting efforts using surgical bioprosthetic valve fracture to improve expansion of the TAVI valve are currently being explored but are not yet routinely practised [24]. In addition, coronary obstruction during ViV-TAVI is of major concern due to its very high mortality rates and the 6‑fold higher incidence in certain bioprosthetic valve types [25, 26]. Bioprosthetic scallop intentional laceration to prevent coronary artery obstruction (BASILICA) has been described for patients at risk for coronary occlusion, and although it has yielded good results in small studies, future study is warranted [27, 28].

In addition, concerns have been raised about the cost-effectiveness of TAVI in intermediate- and low-risk patients [29]. In inoperable patients, TAVI has shown an acceptable incremental cost-effectiveness ratio per quality-adjusted life year [30]. In high- and intermediate-risk patients, the costs-effectiveness of TAVI is consistently less favourable than that of SAVR and is mainly attributed to significantly higher device costs (TAVI ~ $35,132 vs SAVR ~ $6,836), which are not compensated by lower costs during follow-up [30–32]. If intermediate- and low-risk ViV-TAVI patients will require multiple procedures, it is reasonable to question costs-effectiveness in this population. Despite the similar clinical outcomes between ViV-TAVI and redo-SAVR in our and other studies that make ViV-TAVI clinically attractive, ViV-TAVI may not be an economically attractive alternative to redo-SAVR. With the increasing emphasis on the balance of clinical outcomes and costs through value-based healthcare practices, future studies should incorporate cost and cost-effectiveness analyses alongside clinical outcomes in intermediate- and low-risk patients to guide the debate on the possible expansion of TAVI indications from an economic perspective [33].

### Study limitations

Our study should be viewed in the context of the limitations of retrospective observational research with a relatively small sample size. In addition, the intervention strategy to treat the degenerated bioprosthesis was based on the local Heart Team’s decision, and due to the lack of standardised guidelines, there may not have been uniformity in decision-making regarding ViV-TAVI and redo-SAVR across centres.

Furthermore, data on essential variables that are known to influence outcomes in ViV-TAVI (e.g. size and type of implanted valve, and access route) were not available. Additionally, pacemaker implantation and follow-up procedures are not mandatory for data submission to the NHR in redo-SAVR patients, which has resulted in missing values. Differences in NHR definitions of vascular complications between ViV-TAVI and redo-SAVR (vascular complications at 30 days vs major vascular complication) hampered comparability of this outcome in our study.

## Conclusion

Patients with degenerated aortic valve bioprostheses treated with ViV-TAVI or redo-SAVR have similar mortality and morbidity. Prospective randomised trials are needed to strengthen the available evidence.

## Supplementary Information

NHR THI & cardiothoracic surgery Registration Committee members
