# Supplementary material for: Report on outcomes of valve-in-valve transcatheter aortic valve implantation and redo surgical aortic valve replacement in the Netherlands
Source: Neth Heart J. 2021 Aug 9;30(2):106–12. doi: 10.1007/s12471-021-01608-0 (PMC8799804; doi:10.1007/s12471-021-01608-0)
Supplement: Supplementary file 1 — NHR THI & cardiothoracic surgery Registration Committee members [file 12471_2021_1608_MOESM1_ESM.docx]

**ELECTRONIC SUPPLEMENTARY MATERIAL**

NHR THI & cardiothoracic surgery Registration Committee members

| **Registration committee members THI** |  |  |
| --- | --- | --- |
| Amsterdam UMC | M.M. | Vis |
| MUMC+ | S. | Kats |
| Amphia | P. | den Heijer |
| Catharina Ziekenhuis | W.A.L. | Tonino |
| Erasmus MC | P.P.T. | de Jaegere |
| HagaZiekenhuis | C.E. | Schotborgh |
| Isala | V. | Roolvink |
| Leids Universitair Medisch Centrum | F. | van der Kley |
| Medisch Centrum Leeuwarden | F. | Porta |
| Medisch Spectrum Twente | M.G. | Stoel |
| OLVG | G. | Amoroso |
| St. Antonius Ziekenhuis | L. | Timmers |
| Radboudumc | H.R. | Gehlmann |
| UMC Utrecht | P.R. | Stella |
| Universitair Medisch Centrum Groningen | H.W. | van der Werf |
| Amsterdam UMC | J.S. | Lemkes |
|  |  |  |
| **Registration committee members cardiothoracic surgery** |  |  |
| Academisch Medisch Centrum | W.J.P. | Van Boven |
| Academisch Ziekenhuis Maastricht (MUMC) | P. | Segers |
| Amphia Ziekenhuis | S. | Bramer |
| Catharina Ziekenhuis | B.M.J.A. | Koene |
| Erasmus Medisch Centrum | J.A. | Bekkers |
| Haga Ziekenhuis, loc Leyweg | G.J.F. | Hoohenkerk |
| Isala Klinieken  (+ Diac. Meppel) | A.L.P. | Markou |
| Leids Universitair Medisch Centrum | T.J. | Van Brakel |
| Medisch Centrum Leeuwarden | F. | Porta |
| Medisch Spectrum Twente | R.G.H. | Speekenbrink |
| OLVG en St. Lucas | W. | Stooker |
| RadboudUMC | W.W.L. | Li |
| Sint Antonius Ziekenhuis | E.J. | Daeter |
| UMC Utrecht | N.P. | Van der Kaaij |
| Universitair Medisch Centrum Groningen | G. | Mecozzi |
| VU Medisch Centrum | A.B.A. | Vonk |
